# Text-Book of Pharmacology and Therapeutics

**Published:** 1903-03

**Authors:** 


**Text-book of Pharmacology and Therapeutics.** Edited by W. HALE WHITE, M.D. Pp. viii., 1040. Edinburgh and London: Young J. Pentland. 1901.

If it is with a feeling not far removed from aversion that one regards treatises on therapeutics generally, the volume under consideration inspires a sentiment of quite opposite character. Dr. Hale White has taken to himself as co-operators many of the most distinguished authorities in the various departments of pharmacology and therapeutics, both in Great Britain and the United States, and their conjoint labours have produced a work which is a distinct gain to medical literature in the English language. In a work in many sections, written by different authorities, it must happen that some topics seem to be treated more adequately and more satisfactorily than some others, and a due sense of proportion cannot always be obtained by the most strenuous and imperative of editors. Of some of the sections which seem to be handled in a specially satisfactory manner, we would mention the chapter on heart tonics, by Dr. Rose Bradford; that on anæsthetics, by Mr. George Rowell; that on electricity, by Dr. J. H. Bryant; and that on serum therapy, by the late Dr. J. W. Washbourn, written previous to his departure for South Africa. Dr. Washbourn thought that "there is no doubt that the incidence of paralysis in diphtheria has increased since the introduction of antitoxine." This may be, he thought, because "the nervous tissues . . . possess as great, or greater, an affinity than the antitoxine for the toxine, and are consequently not protected by the antitoxine." If this be true, it is possible that some substance may yet be found which will protect against paralysis. Dr. Shoemaker, of Philadelphia, writes about some drugs in the *United States Pharmacopœia* which are not of material therapeutic importance, and the learned editor contributes an useful section upon drugs which are recognised in the Indian and Colonial addendum to the *British Pharmacopœia* of 1898. We need not say that this is well done; but if our *Pharmacopœia* is designed to be co-extensive in use and validity with the British Empire, Calcutta and Cape Town, Melbourne and Montreal should each independently be allowed to supply information and criticism upon the remedies peculiar to their own several countries.

The work is remarkably free from errors; the statement that Nordrach-on-Mendip is on the Quantocks—a venial slip—is one of the very few mis-statements we have noticed. It has been most carefully freed from typographical errors, and is provided with an excellent index. Altogether, the book is a credit to editor, authors, and publishers alike.

